# Molecular Differentiation of *Mycoplasma gallisepticum* Outbreaks: A Last Decade Study on Italian Farms Using GTS and MLST

**DOI:** 10.3390/vaccines8040665

**Published:** 2020-11-09

**Authors:** Andrea Matucci, Elisabetta Stefani, Michele Gastaldelli, Ilenia Rossi, Gelinda De Grandi, Miklós Gyuranecz, Salvatore Catania

**Affiliations:** 1 Mycoplasma Unit—SCT1, Istituto Zooprofilattico Sperimentale delle Venezie, Via San Giacomo 5, 37135 Verona, Italy; amatucci@izsvenezie.it (A.M.); estefani@izsvenezie.it (E.S.); mgastaldelli@izsvenezie.it (M.G.); irossi@izsvenezie.it (I.R.); GDeGrandi@izsvenezie.it (G.D.G.); 2 Institute for Veterinary Medical Research, Centre for Agricultural Research, Hungária körút 21, 1143 Budapest, Hungary; m.gyuranecz@gmail.com

**Keywords:** *Mycoplasma gallisepticum*, GTS, MLST, vaccine, poultry

## Abstract

*Mycoplasma gallisepticum* (MG) infects many avian species and leads to significant economic losses in the poultry industry. Transmission of this pathogen occurs both horizontally and vertically, and strategies to avoid the spread of MG rely on vaccination and the application of biosecurity measures to maintain breeder groups as pathogen-free. Two live attenuated MG vaccine strains are licensed in Italy: 6/85 and ts-11. After their introduction, the implementation of adequate genotyping tools became necessary to distinguish between field and vaccine strains and to guarantee proper infection monitoring activity. In this study, 40 Italian MG isolates collected between 2010–2019 from both vaccinated and unvaccinated farms were genotyped using gene-targeted sequencing (GTS) of the cythadesin gene *mgc2* and multilocus sequence typing (MLST) based on six housekeeping genes. The discriminatory power of GTS typing ensures 6/85-like strain identification, but the technique does not allow the identification ts-11 strains; conversely, MLST differentiates both vaccine strains, describing more detailed interrelation structures. Our study describes MG genetic scenario within a mixed farming context. In conclusion, the use of adequate typing methods is essential to understand the evolutionary dynamics of MG strains in a particular area and to conduct epidemiological investigations in the avian population.

## 1. Introduction

*Mycoplasma gallisepticum* (MG) is one of the four main avian pathogenic *Mycoplasma* species [1]. MG can cause reproductive and/or respiratory disorders, more specifically airsacculitis and other chronic respiratory diseases (CRD) [2]. MG mainly affects turkeys and chickens, but the infection of other avian species, such as quails, geese, guinea fowls, house finches, etc., is also reported [3]. This pathogen causes significant economic losses in the poultry industry due to higher carcass condemnation rates, growth retard, embryonal mortality, and reduced meat and egg production. Transmission of MG can occur horizontally by direct or indirect contact (dust, aerosol, etc.) or vertically through the egg [4]. The maintenance of MG-free breeder flocks is the most adequate method to contain the infection and prevent pathogen spread. One such approach relies on strict biosecurity measures and prompt detection of MG infections.

The antibiotic treatment has proven poorly effective for mycoplasma infection containment as it can only reduce the clinical signs and it is not sufficient for pathogen eradication in chronically infected flocks [4]. In addition, it has been observed for *Mycoplasma synoviae*, which prolonged antibiotic administration results in a progressive increase in the frequency of resistant strains isolation [5].

MG transmission can be reduced by vaccination of the animals with live-attenuated MG strains, which generate mild infections and activate an immune response very similar to that induced by wild-type strains. Vaccination can thus prime the host with both innate and adaptive immunity against subsequent MG infections, leading to efficient pathogen clearance [6]. Three main live attenuated MG vaccine strains have been developed and commercially available: ts-11 (TS-11^®^, Boehringer Ingelheim Animal Health Italia), 6/85 (Nobilis^®^ Mg 6/85, MSD Animal Health), and F-strain (Cevac ^®^ MG F, Ceva Santé Animale). They have shown high efficacy in controlling the spread of MG in chickens but were not demonstrated to be useful in turkey protection [7,8]. Moreover, F-strain is not recommended for use because of its possible virulence in broilers and turkeys [4,9].

Only ts-11 and 6/85 vaccines are licensed in Italy; the use of 6/85 is allowed for layer hens only, while ts-11 can be used also in breeders [10]. However, the use of live-attenuated vaccines as a strategy for MG control in high-density poultry populated areas can complicate achieving early diagnosis and make difficult carrying on outbreak investigations. The correct genotyping of the circulating mycoplasmas is then essential to discriminate field strains from vaccine ones. In addition, appropriate genotyping protocols allow tracking infections and inferring relatedness between strains. A number of different genotyping methods has been published so far, each with specific advantages and disadvantages. DNA fingerprinting techniques [11] are known, for instance, to be labor-intensive and poorly reproducible. Sequence-based methods were developed as gene-targeted sequencing (GTS) for an array of specific variable surface protein genes including cytoadhesin (*mgc2*), which participates in the attachment mechanism of MG to its host [12]. Alternatively, a single-locus sequence typing (SLST) of the intergenic spacer region (IGSR) between the 16S and 23S rRNA genes [13] is described as well. However, these typing methods could be not suitable to infer evolutionary relatedness, albeit they can distinguish different outbreaks and could identify vaccine-related strains. Multilocus sequence typing scheme (MLST) of selected housekeeping genes has been widely regarded as a gold-standard for microbial typing and evolution inference. MG MLST schemes have been recently standardized [14,15] and shown to be capable of revealing genetic relationships between samples. Data generated by MLST can be directly compared between laboratories and easily shared through dedicated databases, such as Bacterial Isolate Genome Sequence Database (BIGSdb) [16].

The Italian public veterinary service, as part of National Health system, is routinely enrolled in surveillance of animal health and food safety. The Istituto Zooprofilattico Sperimentale delle Venezie (IZSVe) is a network of laboratories located in the North East of Italy, a densely populated poultry area (DPPA). Both industrial and backyard animal samples are conferred for diagnostic investigations as well as for local breed conservation programs. 

In this study, a representative set of MG isolates obtained at the IZSVe diagnostic laboratory over years 2010–2019 was analyzed by *mgc2*-GTS and MLST based on a six-housekeeping gene panel. Our results define the scenario of MG infection and evolution in the Italian avian sector and shows the different characteristics and performances of the typing methods used in the description of the genetic interrelations among the isolates.

## 2. Materials and Methods 

### 2.1. Sample Selection and Mycoplasma Isolation

In this study, 40 isolates of *Mycoplasma gallisepticum* were genetically characterized. These isolates were collected from chickens (21), turkeys (14), geese (2), quail (1), and guinea fowls (2), during the years 2010–2019 (Table 1). More than 90% of these samples were of industrial poultry origin, the remaining were backyard or private producer avian samples from different farming areas, mainly from the Italian northern regions (Piemonte, Lombardia, Veneto, Emilia-Romagna). Some strains were collected from the same farm on the same production cycle (e.g., IZSVE/2011/5595-2d and IZSVE/2011/6798/20). Others originated from repeated samplings over short (2 months, e.g., IZSVE/2019/9484 and IZSVE/2019/11886-13) or long (1 year, e.g., IZSVE/2017/514-1f and IZSVE/2018/229-1f) time frames. 

Tracheal swabs collected from both vaccinated and non-vaccinated animals, which displayed typical signs of MG infection, were used for *Mycoplasma* isolation. In order to ensure Mollicutes vitality, immediately after sample collection, the swabs were immersed into 1 mL of transport medium or in a selective medium (Avian *Mycoplasma* Liquid Medium, *Mycoplasma* Experience^®^, Reigate, UK) and maintained at +4 °C until arrival at the laboratory. Each sample was then inoculated into two separate tubes, one containing 2 mL of *Mycoplasma* Experience liquid medium and one containing 2 mL of PPLO (BD Difco^TM^, Worthing, UK) and incubated at 37 ± 1 °C under 5% CO_2_ for 21 days. During this period, the broths were checked daily and, in case a change in color or turbidity was observed, they were inoculated onto an agar plate of Avian *Mycoplasma* Agar (*Mycoplasma* Experience^®^, Reigate, UK) and incubated at the same conditions as above. Samples displaying no changes were all plated at the end of the observational period. All plates were then daily checked for the presence of any “fried-egg” colony [5,17] for up to 7 days. If no colonies were observed, samples were considered negative.

### 2.2. Nucleic Acid Extraction and Species Identification

In order to identify the *Mycoplasma* species, DNA was extracted from all but negative broth samples with Maxwell DNA LEV Blood DNA kit in a Maxwell-16^®^ Instrument (Promega, Milan, Italy) following manufacturer’s instruction. A fragment of the 16S-rDNA gene was then amplified and analyzed by denaturing gradient gel electrophoresis (DGGE) for species identification, comparing the electrophoretic pattern of interest with the one of the MG reference [18].

### 2.3. Vaccine Specific gapA-PCR 

Samples were analyzed for amplification of vaccine strain specific *gapA* sequence via PCR with primers PRUMG32-F/PRUMG36-R [19] (Table S1). The reaction mix was prepared as reported by Evans and collaborators, and amplification was carried out with a BioRad T100 Thermal Cycler (Bio-Rad, Milan, Italy) according to the published thermal profile. The PCR products were loaded and visualized by capillary electrophoresis with the instrument QIAexcel (QIAGEN, Milan, Italy). The expected size of the vaccine specific *gapA* amplicon was 110 bp as confirmed by amplification of ts-11 and 6/85.

### 2.4. Molecular Typing by mgc2-GTS 

A portion of about 300 bp of the *mgc2* gene was amplified with the primers reported in Table S1, following the published protocol [20,21]. Products of amplification were cleaned up using the Performa DTR Ultra 96-Well kit (Edge BioSystems, San Jose, USA) and sequenced in both directions with the same amplification primers using BigDye Terminator v3.1 cycle sequencing kit in a 16-capillary ABI PRISM 3130xl Genetic Analyzer (Thermo Fisher Scientific, Monza, Italy). Sequence data were assembled and edited with SeqScape software v2.5 (Thermo Fisher Scientific, Monza, Italy) or with BioEdit software 7.2.6.1. The assembled *mgc2* sequences were aligned using MEGA 7.0.26 and *mgc2*-type was assigned on the basis of 100% identity with *mgc2* reference sequences, as described in Table 2. An arbitrary color code for each sequence of *mgc2*-type was assigned for a simpler MG genotype definition. 

### 2.5. MG MLST

For MLST analysis, DNA was extracted from culture broths with the QIAamp DNA Mini Kit (Qiagen, Milan, Italy), according to the manufacturer’s indications. Six selected housekeeping genes (*atpG, dnaA, fusA, rpoB, ruvB, uvrA*) were amplified in separate tubes in a 20 μL total volume using the primers listed in Table S1. Each reaction mix contained 10 μL of Sybr Fast Universal Master Mix (2X) (Merck Life Science, Milan, Italy), the specific primer pair at the concentration reported in Beko et al. [14] and 1.5 μL of template DNA. The thermal profile consisted of an initial denaturation/enzyme activation for 5 min at 95 °C followed by 35 cycles comprising a denaturation step at 95 °C for 1 min, primer annealing at 56 °C for 30 s and extension at 72 °C for 1 min. The amplification reaction was performed in a BioRad CFX96 thermal cycler (BioRad, Milan, Italy), acquiring the fluorescence in the FAM/SYBR channel at the end of the extension step. Successful amplification was scored by the presence of a fluorescent sigmoidal curve. The amplified products were then subjected to Sanger sequencing using the same primer set utilized for amplification. New allele sequences and sequence types (STs) found in this study were submitted to the *Mycoplasma gallisepticum* MLST database PubMLST [22] curated by Dr. Ghanem Mostafa (University of Maryland, College Park, MD, USA).

### 2.6. Phylogeny

The minimum spanning trees (MST) presented in this work were constructed with the software Phyloviz 2.0 under Java 1.8.0 environment, implementing the goeBURST algorithm [20]. The MLST profiles were clustered into clonal complexes (CCs) and singletons (Ss) with a dual-level variation cutoff. No clustering cutoff was applied for the full MST analysis of all STs available in PubMLST MG database (August 2020).

Phylogenetic analyses were performed on the sequences of *mgc2* gene and of the MLST housekeeping genes, concatenated in alphabetical order (*atpG-dnaA-fusA-rpoB-ruvB-uvrA*). Upon alignment with the ClustalW algorithm in MEGA 7.0.26, the best fitting maximum likelihood models were identified on the basis of the lowest Bayesian information criterion value. Genetic relations among MG strains were then inferred via a Jukes-Cantor model [23] in the case of *mgc2*, while for MLST concatenates, a Tamura-3 parameter model [24] was selected. In both cases, the rate variation among sites was described by a gamma distribution. Initial trees for the heuristic search were automatically obtained by applying Neighbour-Join and BioNJ algorithms to a matrix of pairwise distances estimated using the Maximum Composite Likelihood (MCL) approach, and then selecting the topology with superior log likelihood value. The bootstrap consensus tree inferred from 1000 replicates was shown to describe the evolutionary history of the taxa analyzed. All positions containing gaps and missing data were eliminated.

### 2.7. Simpson’s Discriminatory Index

The discriminatory power of the different typing schemes was calculated using Simpson’s index of diversity [25] which expresses the probability of two unrelated strains of being characterized as the same type.

## 3. Results

### 3.1. MG Typing with mgc2-GTS

Samples were genotyped by *mgc2*-GTS and grouped for perfect nucleotide alignment with strain reference sequences as described in Materials and Methods. Arbitrary color names were assigned for simplicity in GTS description and results discussion. Genotype aligning with amplified fragment of *mgc2* sequence of vaccine strains 6/85 and ts-11 were then named Orange and Pink, respectively. Phylogenetic relations among the *mgc2* sequences of the MG isolates is represented in Figure S1. The most frequently found color type was pink (17 samples) and included ts-11-like strains, followed by Light blue (13), Green (4), Orange (3), Grey (2) and Black (1). All Orange-type strains were from chickens and vaccine marker gene *gapA* was found in all (3/3) these samples. Pink samples were found in different avian species but only 5 were *gapA*-positive. Four of these isolates were found in vaccinated farms while one came from a farm which vaccination status was not known. These findings indicate the presence of a high level of correlation between the isolate and the vaccination status of the farm of origin (Table 1). Only one Black *mgc2* type from a MG isolated in 2010 from a guinea fowl was observed. The Simpson’s discriminatory index determined for *mgc2*-GTS was 0.70.

### 3.2. MG Typing with MLST

The MG isolates were analyzed with a MLST scheme developed on six selected housekeeping genes [14]. In vitro cultured MG strains showed sufficient genetic stability maintaining the same ST for at least 100 passages (data not shown). As previously reported [14], vaccine strain 6/85 was genotyped as ST14 and ts-11 as ST49. New allele sequences were described in this study: 1 for *fusA* (assigned number 21), 3 for *rpoB* (assigned numbers 22, 23, 24), and 1 for *uvrA* (assigned number 18). In total, 23 STs were described, 9 previously published, and 14 (starting from ST60) of new PubMLST assignment (Table 1). Analysis of the distribution of different alleles composing the ST of this set of samples is reported in Table S2. Among the six genes, *atpG*, *dnaA*, *fusA, ruvB,* and *uvrA* displayed, one or two predominant alleles while *rpoB* appeared as the most variable with allele 15 found with the highest frequency. For this set of samples, the resulting MLST Simpson’s discriminatory index was 0.94.

**Figure 1:**
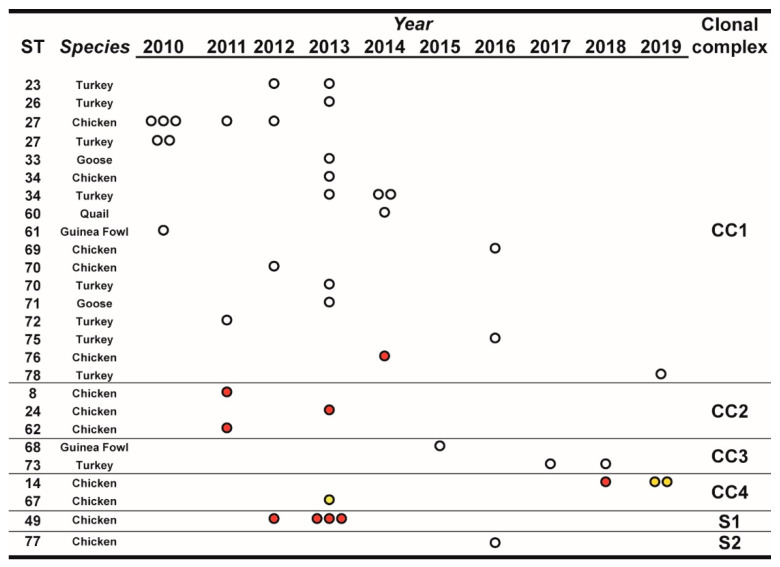
Temporal distribution of sequence types (STs), in relation to species and year of isolation. STs were clustered for clonal complexes (CCs) and singletons (Ss) as described in the text, Section 3.3. The number of dots indicate the number of isolates of the particular ST/year. White dots indicate isolates from non-vaccinated flocks, red dots indicate isolates from vaccinated flocks, and yellow dots indicate isolates from farms with unknown vaccination status.

Over the observation period, the number of isolated MG strains peaked in 2013, and starting from 2015 it showed an apparent declining trend compared to the previous years. Most STs, comprising a large part of the newly defined ones, were isolated only once during the observation period and from a single species (Figure 1). Interestingly, ST27, ST34, and ST70 were isolated from both turkey and chicken. ST14 and ST49 were found only in chicken samples.

### 3.3. Sequence Type Evolution and Phylogeny

**Figure 2:**
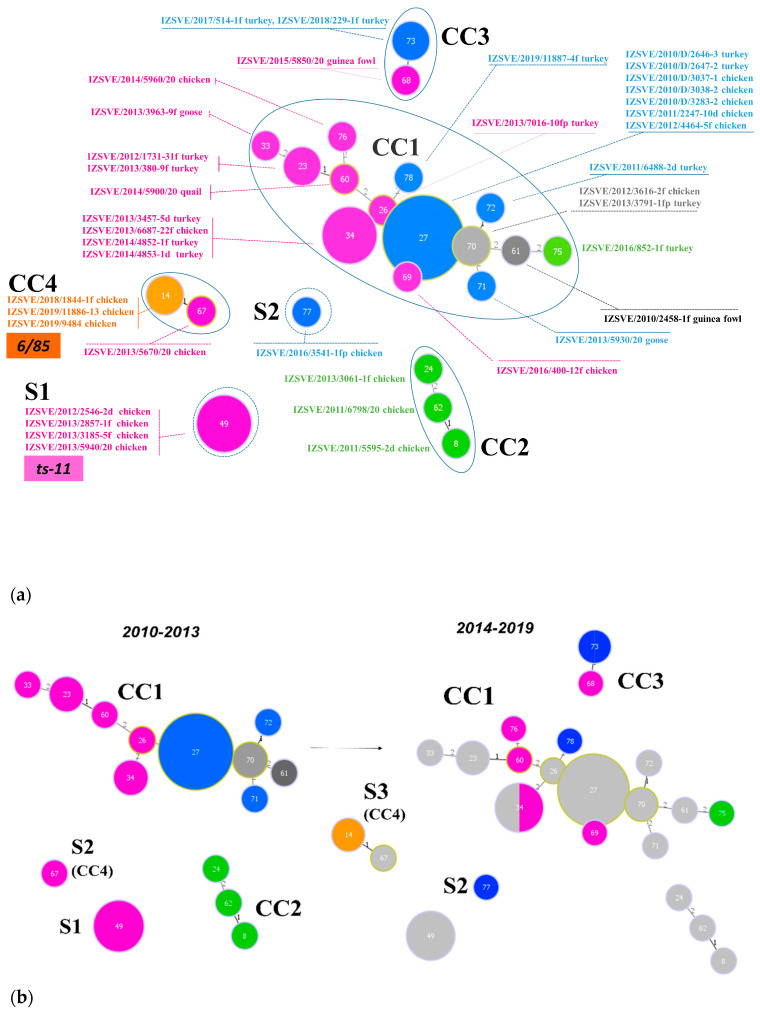
Minimum spanning tree (MST) of the ST with cutoff of 2 differences. Each circle represents a sequence type; the filling and text color represent the respective *mgc2*-GTS genotype. The size of the circle corresponds to the relative number of isolates within that genotype. The numbers inside circles indicate the ST. The numbers over the connecting lines quantify the locus variant levels. (**a**) All samples ST described in this study, including vaccine controls ts-11 and 6/85. CCs and Ss were enclosed by a continuous or dotted line, respectively. (**b**) MSTs of *Mycoplasma gallisepticum* (MG) samples isolated in years 2010–2013 (left) and 2014–2019 (right). Light grey STs on right panel represent 2010–2013 ST footprint in the 2014–2019 year samples.

Inferred evolutionary relationships between all STs found in this study were represented with a minimum spanning tree (MST) from the goeBURST algorithm (Figure 2a). Applying a dual-level variation cutoff, we found four clonal complexes and two singletons. The CC1 was broader with the majority of STs (14 out of 23, 61%) and samples (25 out of 40, 63%). ST27 and ST34 were the most represented types, including almost half of CC1 isolates. CC1 STs were clustered into five *mgc2* types, Pink, Light blue, Grey, Black, and Green. Among these, the first two included the majority of the STs and isolates. No vaccine-related ST was found in CC1. Interestingly, in the years 2010–2013, about 69% of MG strains were assigned to CC1, while in the second half of the observational period (2014–2019), such frequency dropped to about 54%. CC2 samples were isolated during 2010–2013 only, disappearing after 2014 when the CC3 appeared (Figure 1 and Figure 2b). CC2 included 3 different STs (ST24, ST62 and ST8), all belonging to *mgc2* type Green. CC3 grouped ST73 and ST68 of *mgc2* type Light blue and Pink, respectively. The CC4 and S1 included all the *gapA*-positive strains isolated in this study. However, while S1 was homogeneous including solely ST49 *mgc2* type Pink strains, CC4 grouped together three ST14 *mgc2* type Orange and one ST67 *mgc2* type Pink. It is worth noting that no other field sample was assigned to these typing groups. The full MST analysis of our STs, together with all those reported in PubMLST database (Figure S2), suggested a tendential segregation of Italian STs towards the “left branch” of the tree together with other European and Mediterranean samples.

**Figure 3:**
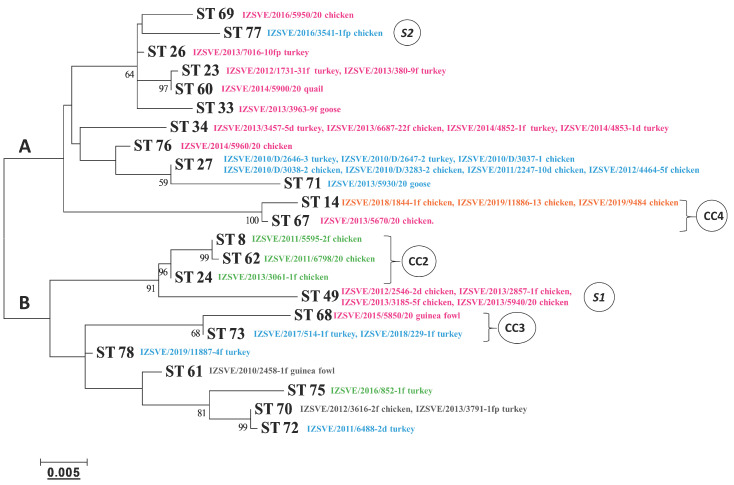
Phylogenetic tree based on the concatenated multilocus sequence typing (MLST) allele sequences of the MG isolates. Clonal complexes (CC) and singletons (S) derived from a dual-level variation cutoff. The letters A and B indicate the two major clades of the dendrogram. Cutoff of bootstrap values was set to 50. Phylogenetic distance bar is represented below.

The phylogenetic tree based on a maximum-likelihood model and relating the concatenate nucleotide sequences of the MLST housekeeping genes is depicted in Figure 3. According to the tree topology, MG strains could be separated into 2 main clades, A and B, including 6/85 ST14 and ts-11 ST49, respectively. Genetically close STs, which formed clonal complexes 2, 3, and 4, were also evident and were supported by high bootstrap values, in good concordance with the MST analysis. 

## 4. Discussion

*Mycoplasma gallisepticum* represents one of the main pathogens in the poultry industry, causing diseases of significant economic impact [26]. MG infection is controlled in the EU under the acts of the European Union Council Directive 2009/158/EC (EU, 2009) and European Commission Decision 2011/214/EU (EU, 2011), which prevent offspring trade from MG positive flocks. To date, successful strategies for mycoplasma spread containment are based on the maintenance of pathogen-free flocks. This can be achieved through the application of strict biosecurity measures coupled with rapid and early identification of infected animal groups. In densely populated poultry farm areas (like the North East of Italy), it is recommended to accurately investigate outbreaks in order to provide useful evidences to the veterinary practitioners that will then be able to improve the biosecurity measures on farm. In addition, preventive use of live vaccines specific for MG (6/85 and ts-11) is considered to be helpful for reducing the negative impacts of MG infection and pathogen shedding. However, the use of vaccine strains could complicate the diagnosis of MG infection at a microbiological, serological, and biomolecular level. A straightforward genotypic classification of circulating MG strains could be fundamental for supporting the strategic choices made in different production lines of both industrial and rural farming. MG isolation and sequencing of specific genetic markers could become useful for single-strain discrimination—including live vaccines—which would allow identifying the outbreaks and carrying out more comprehensive epidemiological investigations. In case of homotypic MG infections, the GTS and MLST analyses could be directly applied to clinical samples [27], shortening the time required for strain identification. However, MG isolation and the production of clonal cultures are in general advisable for carrying out genotype analyses. In this study, 40 different MG samples collected in the northern Italian regions by IZSVe during 2010-2019 were analyzed with a GTS and a MLST protocol. The geographical area investigated in this study is characterized by a high density of poultry farms. Although the majority of the sampled ones applied an intensive livestock production system, our dataset included other zootechnical practices, such as backyard rearing and minor avian species farming.

The high mutation rate of mycoplasma genome results in a pronounced intra-specific variability [28,29] leading to the appearance of multiple different strains. Typing methods based on sequencing of specific genetic markers can prove themselves useful in MG differentiation and typing. It is not surprising that the performances of these protocols depend on the biological function and on the number of the utilized molecular markers. GTS analysis of surface protein genes is reported to be a sensitive and reproducible typing method [12]. However, since these proteins are often involved in host attachment and invasion, their inter-strain variability can be limited. The sequence analysis of cythadesin *mgc2* gene clustered the Italian MG isolates into six main types that were arbitrary color-coded for simplicity. As expected, the assessed discriminatory index appeared relatively low.

As an alternative approach, a six-housekeeping gene MLST scheme [22] was used. Another MLST protocol was recently applied to samples from USA, UK, and Israel [15], and it is available at the same website. The availability of a public database allows data sharing and the adoption of a common ST nomenclature, significantly expanding the power and usefulness of the MLST protocols. In this study, we found previously described STs and newly observed allele combinations and/or allele sequences, which were uploaded to the online database and univocally defined (from ST60 to ST78). Among all, the most variable allele sequence belongs to the *rpoB* gene, with 12 different variants. This gene encodes for the β-subunit of the RNA polymerase, it is known to be a good molecular marker for studying bacterial species evolution [30], and displays the highest diversity index in MLST [14]. Vaccine-associated STs, ST49, for ts-11 and ST14 for 6/85, were found in outbreaks of known vaccinated flocks with no clinical signs or history of *Mycoplasma* field infection. This may indicate that vaccine genotype could be re-isolated and identified by the molecular biology techniques used in this study. Thanks to its discriminatory power, the MLST approach may allow making useful epidemiological evaluations. For example, ST27 (*mgc2* type Light blue) was isolated in 2010 from an industry in the North East area of Italy. In the following 2 years, it was re-isolated not only from the farms related to that same industry, but also from private farms and backyard animals present in the surroundings. ST23 (*mgc2* type Pink) was found in the same turkey farm for 2 consecutive years (2012–2013), while ST34 (*mgc2* type Pink) isolation peaked in 2013 and 2014 in different chicken and turkey farms. Interestingly, a guinea fowl isolate obtained in 2015 (ST68, *mgc2* type Pink) and another one (ST73, *mgc2* type Light blue) isolated twice from the same turkey farm in a 2-year time frame (2017–2018) were clustered together in CC3. These results let us to suppose that CC3 MG strains may represent a recent introduction in Italy, probably through minor avian species or rural poultry. These animals could act as a reservoir of MG with a possible role in the evolution of the circulating strains, confirming previous observations [31]. 

In the Italian context, the *mgc2* GTS genotyping could help differentiating field strains from the vaccine ones, but interpretation is somehow complicated as previously observed by our laboratory [32]. Apparently, through GTS it was achieved a correct identification of all 6/85-like strains, as all Orange samples were isolated from vaccinated animals and tested *gapA*-positive [19]. MLST confirmed the GTS findings, assigning ST14 only to Orange typed Italian strains. 

On the other hand, it is not possible to cluster ts-11-like with *mgc2*-GTS. Pink samples included *gapA*-positive strains and unrelated *gapA*-negative ones. Conversely to Orange samples, MLST analysis assigned different STs to Pink GTS samples and the MST analysis clustered the Pink type strains into three different clonal complexes (CC1, CC3, CC4) and one singleton (S1). The latter was separated from any other Italian STs described in this study because it differed for at least 3 allele changes. All *gapA*-positive ts-11-like strains fell within S1 (ST49). It should be noted, however, that other works described the existence of ST49-close types, like ST48 and ST50 [14]. The ST48 strain, with a different *fusA* allele compared to ST49, was described in a particular Italian isolate obtained by IZSVe in 2013. The ST50 differed from ST49 for the *rpoB* sequence and it was found in K6216D strain, a described ts-11-like strain that displayed higher virulence in chickens [33]. ST48 and ST50 were both *mgc2* typed as Pink (data not shown). According to both the maximum likelihood and the full MST tree, the CC2 ST8, ST24, and ST62 are in close genetic relationship with ST49. ST8 and ST62 were two different strains isolated from the same chicken layer farm-house in 2011, a plausible occurrence in a multiage housing system. ST24 was a chicken isolate, collected in 2013 from a different poultry sector than the previous two. In terms of *mgc2*-GTS, CC2 samples resulted being type Green, phylogenetically distant from Pink isolates (Figure S1). Interestingly, all of the CC2 samples derived from vaccinated farms, but the isolated and typed strains did not test positive for *gapA*. However, in the maximum likelihood tree, CC2 STs significantly clustered within the same clade with S1 (ST49).

MST analysis indicated that all 6/85-like ST14 were clustered in a single complex (CC4) with ST67. This latter strain was isolated in 2013 from a backyard chicken; it was GTS type Pink and *gapA*-positive. These observations, at first sight, would lead us suspecting a ts-11-like strain, but the MLST analysis and the genetic comparison indicated closer correlation with 6/85. It is noticeable that ST67 is genetically distant from the majority of the circulating strains (CC1) and may have originated from ST14 strain. Apparently, no other strain clustered in CC4 in the following years, which may indicate that either diffusion or evolution from ST67 did not occur. This hypothesis needs to be confirmed by a constant field monitoring.

The majority of samples from vaccinated farms display an ST that did not cluster into CC1, letting us to suppose that the vaccination could not only decrease the probability of MG infection to establish but also influence the genotype of the spreading MG population. Starting from 2014, we have been noticing a more and more decreased number of outbreaks while, at the same time, an increase in previously undescribed STs isolation (ST60 to ST78). In the period 2014–2019, only eleven samples (excluding ST14) were isolated, eight of which were new assignment STs. These observations could be explained by the increased farm biosecurity measures adopted to face other, MG-independent factors, such as H7N7 HPAI epidemic in Italy [34] in late summer 2013, or eggshell apex abnormality outbreaks [17,35]. 

Core genome MLST was recently described [36] for an in depth analysis of MG strains, but it cannot be routinely applied to a broad set of samples. The development of a MLST-s scheme (surface integrated MLST) that integrates housekeeping genes typing with surface protein encoding genes (as *mgc2*), could be very useful to increase the discriminatory power for local epidemiological studies, as recently described for *Mycoplasma hyorhinis* [37] and could be evaluated also for future studies on avian mycoplasmas. 

## 5. Conclusions

The biomolecular characterization of MG outbreaks is essential to identify circulating strains and differentiate vaccine isolates from field ones. In this study, on a total of 40 Italian samples isolated mainly from chickens and turkeys over the last decade, we found that Pink type was the most prevalent when using *mgc2*-GTS, while the CC1 was the most prevalent when using MLST. This latter complex included also strains isolated from minor avian species, suggesting a possible role of these birds in MG diffusion and evolution. Vaccine sequence types are not part of CC1 and segregate into a different cluster or singleton. In our dataset, both GTS and MLST group all 6/85-like strains into a single type. However, only MLST could properly cluster ts-11-like strains into a unique ST. MG genotypes closely related to the vaccine ones were found in the observational period and should be deeply studied in the future. The use of molecular methods based on structural or housekeeping genes (or in combination) is essential to understand the dynamic of evolving MG genotypes in a particular territory, improving the active monitoring of the circulating strains in the industrial population for which live vaccine treatment is licensed. The routine application of genetic screening plans on MG-vaccinated farms could be of a great importance in the appropriate use of 6/85 and ts-11 strains. 

This study confirms the importance of genotyping MG strains coming from outbreaks in both industrial and rural poultry for pursuing genetic investigations using GTS as isolate differentiation method. At the same time, it becomes clear the need of carrying out fine epidemiological studies using MLST, which allows keeping track of evolving genotypes in both poultry vaccinated and non-vaccinated farms, resulting in an efficient monitoring system of vaccine and field strains circulation. 

## Figures and Tables

**Table 1:** Description of MG isolates analyzed in this study and genotyping results.

Sample ID	Farm ^1^	Year	Sp ^2^	Vax ^3^	*mgc2*-GTS ^4^	*gapA* ^5^	MLST 6-alleles						ST
							*atpG*	*dnaA*	*fusA*	*rpoB*	*ruvB*	*uvrA*	
IZSVE/2011/5595-2d	5-AA3	2011	C	y	Green	N	3	2	10	9	1	1	8
IZSVE/2018/1844-1f	6-T1	2018	C	y	Orange	P	5	8	6	3	5	10	14
IZSVE/2019/9484	1-O1	2019	C	n.d.	Orange	P	5	8	6	3	5	10	14
IZSVE/2019/11886-13	1-O1	2019	C	n.d.	Orange	P	5	8	6	3	5	10	14
IZSVE/2012/1731-31f	7-R1	2012	T	n	Pink	N	8	1	1	5	2	13	23
IZSVE/2013/380-9f	2-R1	2013	T	n	Pink	N	8	1	1	5	2	13	23
IZSVE/2013/3061-1f	8-G1	2013	C	y	Green	N	8	1	10	9	1	1	24
IZSVE/2013/7016-10fp	2-I1	2013	T	n	Pink	N	8	1	16	4	2	12	26
IZSVE/2010/D/2646-3	1-P1	2010	T	n	L-blue	N	8	1	16	15	1	12	27
IZSVE/2010/D/2647-2	1-P2	2010	T	n	L-blue	N	8	1	16	15	1	12	27
IZSVE/2010/D/3037-1	1-P3	2010	C	n	L-blue	N	8	1	16	15	1	12	27
IZSVE/2010/D/3038-2	1-P4	2010	C	n	L-blue	N	8	1	16	15	1	12	27
IZSVE/2010/D/3283-2	1-P3	2010	C	n	L-blue	N	8	1	16	15	1	12	27
IZSVE/2011/2247-10d	V1	2011	C	n	L-blue	N	8	1	16	15	1	12	27
IZSVE/2012/4464-5f	BY	2012	C	n	L-blue	N	8	1	16	15	1	12	27
IZSVE/2013/3963-9f	T1	2013	G	n	Pink	N	8	5	16	5	2	13	33
IZSVE/2013/3457-5d	P4	2013	T	n	Pink	N	8	5	16	7	2	12	34
IZSVE/2013/6687-22f	9-I1	2013	C	n	Pink	N	8	5	16	7	2	12	34
IZSVE/2014/4852-1f	1-I1	2014	T	n	Pink	N	8	5	16	7	2	12	34
IZSVE/2014/4853-1d	1-R1	2014	T	n	Pink	N	8	5	16	7	2	12	34
IZSVE/2012/2546-2d	3-V2	2012	C	y	Pink	P	13	11	14	8	9	2	49
IZSVE/2013/2857-1f	4-B1	2013	C	y	Pink	P	13	11	14	8	9	2	49
IZSVE/2013/3185-5f	3-V3	2013	C	y	Pink	P	13	11	14	8	9	2	49
IZSVE/2013/5940/20	4-B2	2013	C	y	Pink	P	13	11	14	8	9	2	49
IZSVE/2014/5900/20	V2	2014	Q	n	Pink	N	8	1	1	5	2	12	60
IZSVE/2010/2458-1f	BY	2010	GF	n	Black	N	2	1	16	17	2	5	61
IZSVE/2011/6798/20	5-AA3	2011	C	y	Green	N	3	3	10	9	1	1	62
IZSVE/2013/5670/20	BY	2013	C	n.d.	Pink	P	5	8	14	3	5	10	67
IZSVE/2015/5850/20	2-T2	2015	GF	n	Pink	N	8	1	8	17	20	12	68
IZSVE/2016/5950/20	P5	2016	C	n	Pink	N	8	13	16	6	1	12	69
IZSVE/2012/3616-2f	1-AA4	2012	C	n	Grey	N	2	1	16	15	1	5	70
IZSVE/2013/3791-1fp	1-I3	2013	T	n	Grey	N	2	1	16	15	1	5	70
IZSVE/2013/5930/20	L1	2013	G	n	L-blue	N	2	5	16	15	1	12	71
IZSVE/2011/6488-2d	1-E4	2011	T	n	L-blue	N	2	1	4	15	1	5	72
IZSVE/2017/514-1f	1-F1	2017	T	n	L-blue	N	8	1	8	17	20	5	73
IZSVE/2018/229-1f	1-F1	2018	T	n	L-blue	N	8	1	8	17	20	5	73
IZSVE/2016/852-1f	2-R2	2016	T	n	Green	N	2	1	8	22	2	5	75
IZSVE/2014/5960/20	4-B3	2014	C	y	Pink	N	8	1	21	23	2	12	76
IZSVE/2016/3541-1fp	2-R3	2016	C	n	L-blue	N	5	1	16	6	2	18	77
IZSVE/2019/11887-4f	1-F1	2019	T	n	L-blue	N	8	1	16	24	2	5	78

^1^ Farm: industry and farm [industry number code]-[Farm alphanumerical code] or backyard sample (BY), absence of industry number code identify private farmer. ^2^ Sp: avian species abbreviated as C (chicken), T (turkey), G (goose), GF (guinea fowl), Q (quail). ^3^ Vax: history of farm vaccine treatment, y: vaccinated; n: not vaccinated; n.d.: not declared. ^4^ *mgc2*-GTS: *mgc2* gene-targeting sequencing genotype color-coded as in Table 2 (Light blue abbreviated as L-blue). ^5^ *gapA:* PCR for vaccine-specific *gapA* target amplification (P: positive, N: negative).

**Table 2:** *mgc2*-GTS color-type reference strains included in the present study.

*mgc2*-GTS Type	Reference Strain	GenBank No.
Pink	MG TS-11	JQ770175.1
Orange	MG 6/85	JQ770178.1
Light blue	IZSVE/6344/3	KM107803
Green	ATCC 15302 (S6)	KF874282.1
Violet	NTCC 10115 (PG31)	AY556239.1
Grey	UHP-1	AY556297.1
Black	IZSVE/2911/21	KM107806

## Supplementary Materials

The following are available online at https://www.mdpi.com/2076-393X/8/4/665/s1. Figure S1. Phylogenetic tree based on the *mgc2* sequence of the MG isolates. Sample name and host are reported in font color accordance with the relative *mgc2* type. Cutoff of bootstrap values was set to 50. Phylogenetic distance bar is represented below; Figure S2. Full MST tree MG MLST 6-genes. Dispersion of full MST of all described six-alleles scheme ST profiles with PubMLST database updated to August 2020. Red circles are Italian ST described in this study, green ST the nodal points, and in light grey, other known STs; Table S1. Primer sequences and expected amplicon size (bp) of the PCR protocols described in this study; Table S2. Allele distribution into sequence type of Italian samples analyzed.
