# Supplementary material for: Molecular Differentiation of Mycoplasma gallisepticum Outbreaks: A Last Decade Study on Italian Farms Using GTS and MLST
Source: Vaccines (Basel). 2020 Nov 9;8(4):665. doi: 10.3390/vaccines8040665 (PMC7712042; doi:10.3390/vaccines8040665)
Supplement: Supplementary file 1 [file vaccines-08-00665-s001.pdf]

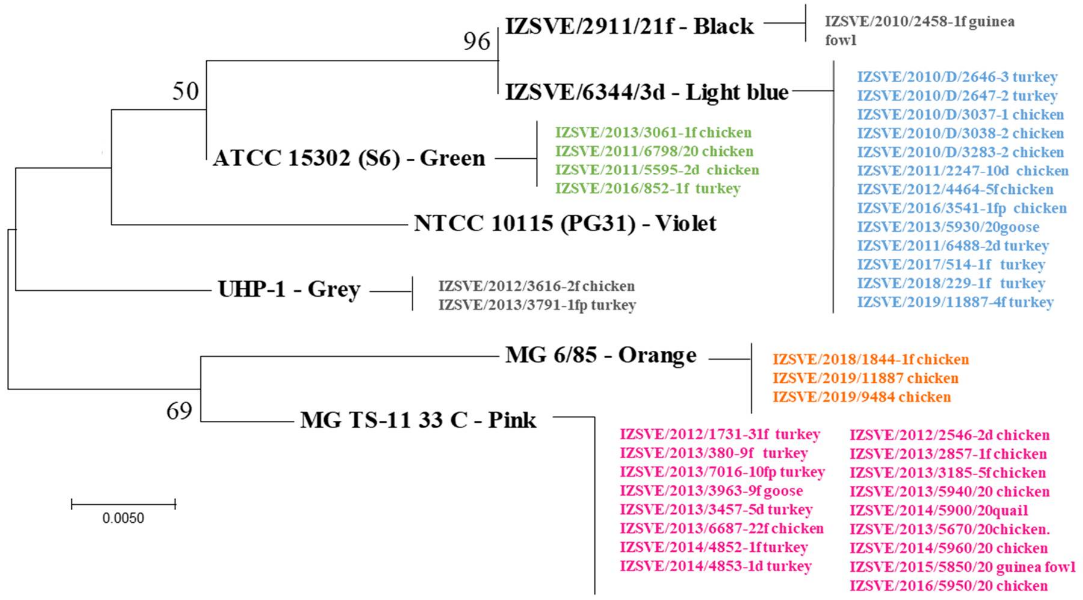

Figure S1. Phylogenetic tree based on the *mgc2* sequence of the MG isolates. Sample name and host are reported in font color accordance with the relative *mgc2* type. Cutoff of bootstrap values was set to 50. Phylogenetic distance bar is represented below.

Table S1. Primer sequences and expected amplicon size (bp) of the PCR protocols described in this study.

Gene Target	Primer Forward (5′->3′)	Primer Reverse (5′->3′)	PCR Amplicon Size (bp)
gapA	GAACTGATTTTGGAA GCGTAAGA	CCGTTATATCATCCTGAAT CTCCT	110
mgc2	CGCAATTTGGTCCTA ATCCCCAACA	TAAACC CACCTCCAGCTTTATTTC C	237–300
atpG	TGGAACTAAACTAA ATTCGTTTTTAAGA	TAGCATACTCACACACTTT GGATTCA	395
dnaA	GAGCGTCAAAAATT ATTCCCAGA	TTACGAATATCGCCTTCAT CAAA	461
fusA	CAGTAGCAGTATTAG ATGCCCAAATG	TAGTAGGGATCTGTACTTC TTCACCAA	597
rpoB	GTTAATGCTTAAAGA ACAACTTGATTTATT	GGTTAATTGGTGCGTGTTA AAGAA	562
ruvB	CAACGACAATGTATG GCAGGAT	AAACAATCAATTCCACTTA TTAGTGAAA	388
uvrA	TTTACCAATCTTAAT GTGAATAAAGCC	CCGTTCCCTGGGTGGAGTT	536

Table S2. Allele distribution into sequence type of Italian samples analyzed.

MLST Target	Observed Number of Allele Variants (Number of Known Variants [1])	Prevalent Allele Number [2] (Number of Samples)
atpG	5 (24)	8 (23). 2 (6)
dnaA	7 (22)	1 (23), 5(6)
fusA	8 (27)	16 (20), 14 (5)
rpoB	12 (33)	15 (11), 3 (4), 5 (4), 7 (4), 8 (4), 17 (4)
ruvB	5 (24)	1 (15), 2 (14)
uvrA	7 (24)	12 (17), 5(8)

[1] Based on https://pubmlst.org/mgallisepticum/ updated on August 2020. [2] Allele sequence present in >50% of samples.

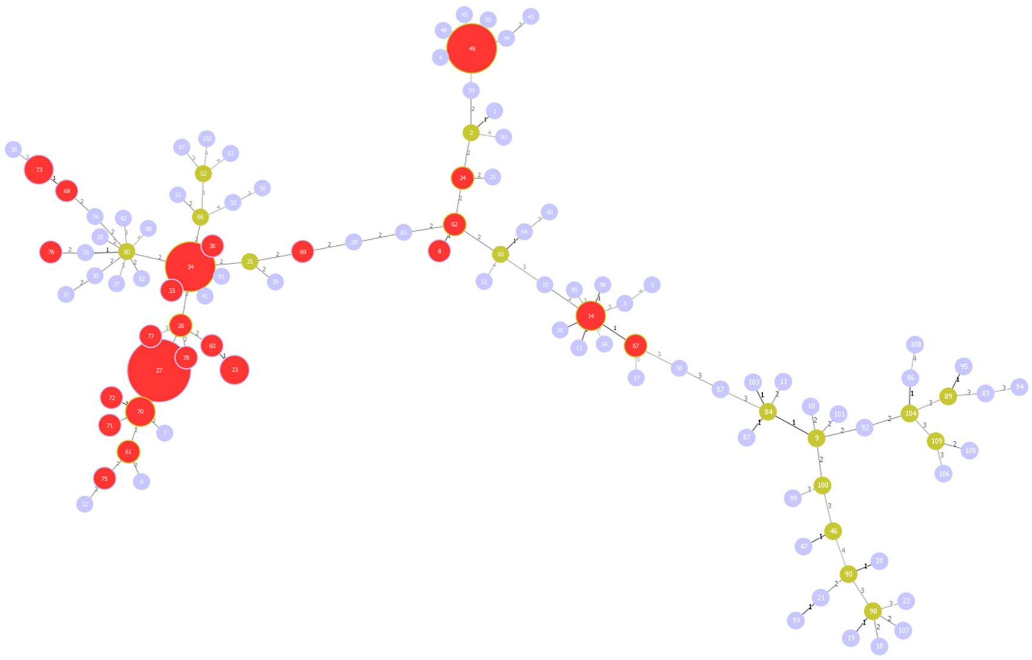

Figure S2. Full MST tree MG MLST 6-genes. Dispersion of full MST of all described six-alleles scheme ST profiles with PubMLST database updated to August 2020. Red circles are Italian ST described in this study, green ST the nodal points, and in light grey, other known STs.
